# Ultrasensitive Chemical Detection Using Integrating Cavity-Enhanced Raman Spectroscopy

**DOI:** 10.1021/acs.analchem.5c07227

**Published:** 2026-06-05

**Authors:** Thomas Z. Moore, Joel N. Bixler, Brett H. Hokr, John D. Mason, Edward S. Fry, Vladislav V. Yakovlev

**Affiliations:** † Texas A&M University, College Station, Texas 77843, United States; ‡ Southwest Research Institute, 6220 Culebra Rd, San Antonio, Texas 78238, United States

## Abstract

Raman spectroscopy is a powerful analytical technique used for molecular detection, identification, and characterization, but its broader utility has been limited by the intrinsically weak spontaneous Raman scattering intensity. In this work, we demonstrate significant Raman signal enhancement using a novel high-performance integrating cavity constructed with newly developed Lambertian materials exhibiting exceptionally high reflectivity. Cavity ringdown measurements yield a peak average reflectivity of 99.943 ± 0.0004% at 610 nm. Raman measurements of bulk methanol, magnesium sulfate, and glycine demonstrate μmol sensitivity using a compact, fiber-coupled 405 nm diode laser delivering 17 mW of optical power. Additionally, limit-of-detection studies performed using a 532 nm diode-pumped solid-state laser at 150 mW demonstrate nanomole-level sensitivity for two common polycyclic aromatic hydrocarbons—benzo­[a]­pyrene and pyrene. These findings establish integrating cavity-enhanced Raman spectroscopy as a promising approach for compact, high-sensitivity systems in medical, environmental, industrial, and space-based applications.

## Graphical abstract

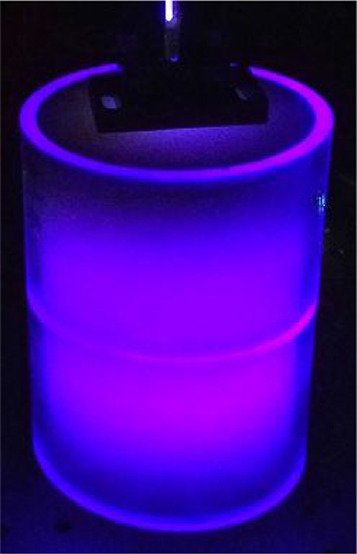

Raman spectroscopy has long been recognized as a powerful tool for the detection, identification, quantification, and characterization of molecular compounds across research laboratory and industrial settings. It has contributed significantly across many diverse fields ranging from environmental monitoring and industrial processes to pharma and medicine and aerospace and defense^1,2^ The technique is particularly attractive because it is nondestructive, with little to no sample preparation required. The emergence of the laser has greatly increased the practicality of Raman spectroscopy as an analytic tool. The availability of today’s compact low-power high-quality lasers at relatively low cost has fueled increased interest in compact Raman instruments. However, the intrinsically weak Raman scattering cross-section continues to be a primary limitation.^3^

The inelastic scattering of light by a molecules was originally theorized by Adolf Smekal^4^ in 1923 and experimentally observed by C. V. Raman and K. S. Krishnan,^5^ and independently by Grigory Landsberg and Leonid Mandelstam,^6^ in 1928. Raman scattering refers to light that is inelastically scattered from a molecule acquiring a frequency shift relative the incident light. This frequency shift is directly related to the molecular modes of a molecule, providing information about the molecular structure, symmetry, and chemical bonds. Raman spectroscopy exploits inelastic scattering to enable quantitative analysis of molecular compounds with a high degree of specificity.^1–3^ While both vibrational and rotation modes are inelastically scattered, we are presently concerned with only the vibrational modes. Unfortunately, Raman scattering is an inherently inefficient process with typical cross sections around 10^–29^ cm^2^ per molecule. By contrast, typical Rayleigh scattering and absorption cross sections are of the order of 10^–26^ and 10^–20^ cm^2^ per molecule, respectively.^7^ The weak Raman signal return has historically limited the widespread use of Raman spectroscopy.^3,7,8^

Several approaches have been developed to enhance the Raman signal. Coherence-based techniques, such as stimulated Raman spectroscopy,^9,10^ coherent anti-Stokes Raman spectroscopy,^11^ and coherent Stokes Raman spectroscopy,^12^ can achieve signal enhancements of 10^4^–10^5^. However, these techniques require multiple, precisely tuned laser frequencies, careful timing control, and *a priori* knowledge of the target analyte. In addition, the strong coherence often leads to line width distortion, complicating the spectral analysis. More recently, resonance enhancement techniques have become popular, including resonance Raman spectroscopy (RRS),^13,14^ surface-enhanced Raman spectroscopy (SERS),^15–20^ and tip-enhanced Raman spectroscopy (TERS).^21–23^ Enhancements of 10^5^ to 10^8^ or more have been reported using resonance enhancement techniques. While SERS and TERS use plasmonic resonances for Raman enhancement, RRS enhancement occurs near the first electronic state. RRS enhancement is unique to each molecular mode thereby distorting the Raman spectra, whereas plasmonic effects in SERS and TERS can perturb the molecular structure shifting the vibrational frequency and distorting the Raman line width complicating the spectral analysis.

Integrating cavity-enhanced Raman spectroscopy (iCERS) is a recently developed technique that exploits the unique properties of an integrating cavity to achieve Raman signal enhancement without introducing spectral distortion. Here, we demonstrate a novel approach to enhance spontaneous Raman scattering. Multiple reflections within the cavity result in repeated light–sample interactions, substantially enhancing the Raman signal. Using this approach, we demonstrate several orders of magnitude Raman signal enhancement is achievable relative to conventional confocal techniques, along with nanomole limit of detection.

## Integrating Cavity Enhancement of Raman Scattering

The concept of using an integrating cavity for spectroscopy was first proposed by Paul Elterman in 1970 to mitigate optical scattering in absorption spectroscopy.^24^ Whereas Elterman was seeking to measure absorption independent of optical scattering, the iCERS technique leverages reflections within a cavity to significantly increase the number of light–sample interactions. The enabling technology of the iCERS technique is a high-performance integrating cavity (HPIC) fabricated from highly reflective Lambertian materials, such as those developed by Fry, Yakovlev et al.^25–27^ This novel Lambertian material has enabled a variety of optical applications, including absorption spectroscopy, fluorescence enhancement, integrating cavity ring-down spectroscopy, and iCERS.^28–36^

### Integrating Cavity

An integrating cavity is an optical device with an enclosed volume in which incident light is spatially integrated, or uniformly distributed throughout. The first theoretical analysis of a cavity with diffuse reflecting walls was published by William Sumpner^37^ in 1892 and experimentally realized by Friedrich Richard Ulbricht^38^ in 1900. Since then, integrating cavities have been used extensively for radiometric measurements ever since.^39^ Recent advancements in high-performance diffuse reflective materials, such as those fabricated from fumed silica, have enabled integrating cavity enhancement.

Cavity-enhanced Raman spectroscopy exploits high-finesse cavities^40,41^ or multipass (Herriott) cells^42^ to enhance the Raman return from gas samples. Similarly, iCERS exploits repeated reflections and cavity buildup as light circulates within the cavity. The cavity buildup, or cavity multiplier, is

1 $$M=\frac{\rho }{1-\mathrm{\rho ̅}},\;\mathrm{\rho ̅}=\rho \left(1-f\right)$$

where *ρ* is the material reflectivity, *ρ̅* is the average cavity reflectivity, and *f* is the port fraction loss, i.e., the ratio of the port area to the total cavity surface area. Cavity multipliers of 250–1500 are typical for HPICs, whereas values of 10–30 are common for commercial integrating cavities. The surface radiance, *L*, is

2 $$L=\frac{\phi_{0}}{\pi S}M$$

with units of W·m^–2^·sr^–1^, where *ϕ*_0_ is the input flux and *S* is the cavity surface area. For a given flux, radiance decreases with increasing cavity size while the input flux is uniformly distributed across the cavity surfaces.

There are several key aspects of integrating cavities that are useful for understanding their unique properties. While a more complete theory of integrating cavities can be found elsewhere,^39,43–45^ the focus here is on the properties relevant to integrating cavity enhancement. For an empty cavity of volume *V* and surface area *S*, assuming uniform light distribution, the temporal response to a short pulse is an exponential decay^39,46^ with a characteristic time

3 $$\tau =\mathrm{n̅}\mathrm{t̅}=\frac{\mathrm{n̅}\mathrm{d̅}}{c}$$

where *t̅* and *d̅* represent the average time and distance between reflections, respectively, and *n̅* = 1/ln­(*ρ̅*) is the average number of reflections. On exit, the effective optical path length is

4 $$l_{eff}=\mathrm{n̅}\mathrm{d̅},\mathrm{d̅}=\frac{4V}{S}$$

where average distance between reflections is proportional to the ratio of the cavity volume to the surface area.^46^ The average cavity reflectivity is

5 $$\mathrm{\rho ̅}=\exp\left(-\frac{4}{c\tau }\frac{V}{S}\right)$$

The characteristic time, *τ*, can be obtained through a direct ringdown measurement from which the average cavity reflectivity can be obtained.

### iCERS Enhancement

The iCERS approach achieves signal enhancement through several mechanisms, including repeated light–sample interactions via multiple reflections, cavity buildup, and light collection over the full 4*π* solid angle. These interactions are further enhanced because the cavity has a much larger number of participating molecules, and each reflection provides repeated opportunities for light–molecule interaction. Within the cavity, the incident, Rayleigh-scattered, and Raman-scattered light all circulate; however, stimulated Raman scattering remains negligible due to the random nature of the interactions and the inherently weak Raman scattering cross-section. Thus, the observed enhancement is dominated by increased photon interaction with a larger number of molecules and efficient collection over the 4*π* solid angle.

The Raman scattering intensity per molecule is

6 $$I_{sc}\propto I_{0}\underset{r}{\sum }{\omega_{s}}^{4}\frac{d\sigma_{r}}{d\Omega };\;\omega_{s}=\omega_{0}-\omega_{r}$$

where *ω*_*s*_ is the stokes frequency, *ω*_0_ is the pump laser frequency, *ω_r_* is the frequency of the *r*th vibrational mode, and *dσ*/*d*Ω is the differential Raman cross-section. In most Raman experiments, the signal arises from an ensemble of *N* molecules, making the total Raman scattering proportional to *I*_0_ *N*. Consequently, the overall Raman signal depends on the number of photons interacting with the molecular ensemble.

In conventional spontaneous confocal Raman spectroscopy (SCRS), an objective lens focuses the excitation light onto the sample, generating a high-intensity focal spot with a high degree of spatial resolution. SCRS leverages the increased excitation intensity within the confocal volume to obtain a strong Raman signal, although the number of molecules within this focal region is relatively small.

In contrast, for iCERS, the intensity on the sample is proportional to the radiance, *L*, in the cavity and is much lower than SCRS; however, the number of participating light–molecule interactions is significantly greater. Transmitted and elastically scattered light can interact with other molecules with an interaction probability defined as *n_int_* (*n̅*) =*η_sv_n̅*, where *η_sv_* is the probability that the reflected light reinteracts with the sample. The Raman scattering intensity within an integrating cavity can be described as,

7 $$I_{sc}\propto \left(I_{0}N_{0}+4\pi LN_{mol}n_{int}\right)\underset{r}{\sum }{\omega_{s}}^{4}\frac{d\sigma_{r}}{d\Omega };\;n_{int}\left(\mathrm{n̅}\right)=\eta_{sv}\mathrm{n̅}$$

Here, the *I*_0_ *N*_0_ term accounts for light directly incident upon the sample, while the 4*πLN_mol_n_int_* term accounts for reflected light that interacts with the sample. The interaction term can be complex, as it depends on the sample size, spatial distribution, and optical properties of the sample. Sample absorption reduces the average number of reflections and therefore diminishes the overall Raman signal. Experimentally, no significant difference has been observed between the Raman signal obtained with excitation incident directly on the sample and that measured without direct incident excitation, i.e., *I*_0_ *N*_0_ = 0.

For a given laser power, the enhancement of iCERS relative to SCRS is expressed as the ratio of their respective scattering intensities,

$$\mathrm{Enhancement}=\frac{I_{sc-iCERS}}{I_{sc-SCRS}}≅\frac{I_{0_{iCERS}}N_{0_{iCERS}}+4\pi LN_{iCERS}n_{\mathrm{int}}}{I_{0_{SCRS}}N_{SCRS}}$$ 8

In many cases where the excitation light is directly incident on the sample, the products $I_{0_{iCERS}}N_{0_{iCERS}}\,\;\;and\,\;\;I_{0_{SCRS}}N_{SCRS}$ are compable. Although the enhancement factor generally exceeds unity under typical operating conditions, this is most readily demonstrated when the same objective used for SCRS is employed in the iCERS configuration, such that $I_{0_{iCERS}}N_{0_{iCERS}}=I_{0_{SCRS}}N_{SCRS}$ . Furthermore, the enhancement can be increased by using laser powers that exceed the photodamage threshold of SCRS, as the excitation light is distributed throughout the cavity, resulting in a substantially lower irradiance at the sample and thereby reducing the risk of photodamage.

## Experimental Section

The integrating cavity reflective performance was determined from the characteristic time, obtained by fitting the exponential decay of the ringdown signal. Short, nanosecond-range laser pulses from an OPOTek Opolette HE 355 LD + UV laser (wavelength range: 248–1080 nm) were used in these measurements. The ringdown signal was detected using a ThorLabs DET10A high-speed silicon photodetector and captured on a Tektronix DPO72004C digital oscilloscope.

Comparative Raman measurements were performed on background air, methanol (MeOH), and magnesium sulfate (MgSO_4_) using both iCERS and conventional SCRS techniques. Identical experimental configurations were employed for both measurement modes, differing only by the coupling approach—either a microscope objective or the HPIC (Figure 1).

**Figure 1:**
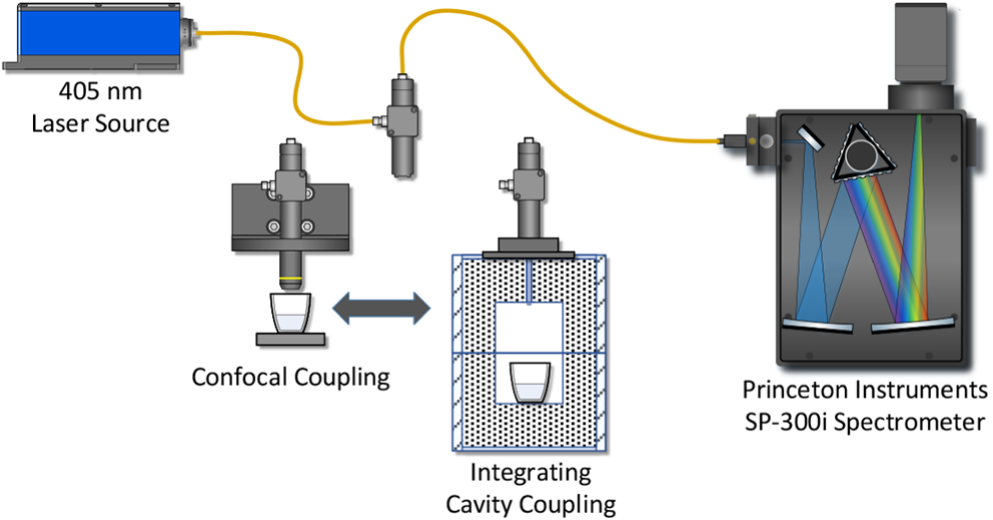
Schematic of comparative Raman testing between iCERS and SCRS. A 405 nm laser (30 mW) is delivered through a single-mode optical fiber to a long-pass dichroic beam splitter, coupling light through either a microscope objective or an HPIC. Samples are placed in a clean fused-silica crucible for measurement. Raman Stokes-scattered light is collimated, transmitted through the dichroic and an additional long-pass filter, then focused into a 400 μm multimode fiber coupled to a Princeton Instruments SP-300i spectrometer. The optical configuration is identical for both systems except for the sample-coupling method. Adapted from author’s Dissertation: *Integrating Cavity Enhanced Vibrational Spectroscopy for Planetary Exploration*, Texas A&M University, 2025.^47^

A 405 nm, 30 mW laser source delivered through a single-mode optical fiber (ThorLabs SM400) was directed onto a long-pass dichroic beamsplitter (Semrock Di03-R405-t3-12.5 × 1.1-D) and subsequently coupled to the sample. The Raman-scattered light was collected and transmitted through the same dichroic beamsplitter, followed by an additional long-pass filter (Semrock LP02-407RU-12.5D). The filtered light was focused into a 400 μm-core multimode optical fiber (ThorLabs FT400UMT) and transmitted to a Princeton Instruments SpectraPro-300i spectrometer. The out-coupled light was focused through a 50 μm entrance slit and collimated onto a 1200 line mm^–1^ grating with a 300 nm blaze. Raman spectra were recorded with a Princeton Instruments 1340 × 100 e2v CCD detector cooled to −45 °C.

Comparative Raman spectral measurements of laboratory air were reported by Texas A&M University (TAMU).^48^ Bulk samples of MeOH (1 mL, CAS 67-56-1) and MgSO_4_ crystals (520 mg, CAS 7487-88-9) were measured by Southwest Research Institute (SwRI). A *bulk sample* is defined as an unprocessed, unoriented specimen that has not been optimized for sample size or distribution. Each sample was placed in a clean fused silica crucible and measured under identical conditions for both experimental configurations. For confocal measurements, an Olympus PLN Plan 10×/0.25 NA achromatic objective was used; for iCERS, a 3 mm fused silica lightguide coupled light into and out of the HPIC.

### Integrating Cavity Design

Two fumed-silica-based HPICs were used in the experimental measurements presented here. One cavity was fabricated at TAMU, as described by Bixler et al. and Cone et al.,^29,30^ while the other was fabricated at SwRI. Both HPICs have the same internal right-cylindrical cavity dimensions (50.8 mm diameter × 50.8 mm height) in a split-cavity design (Figure 2).

**Figure 2:**
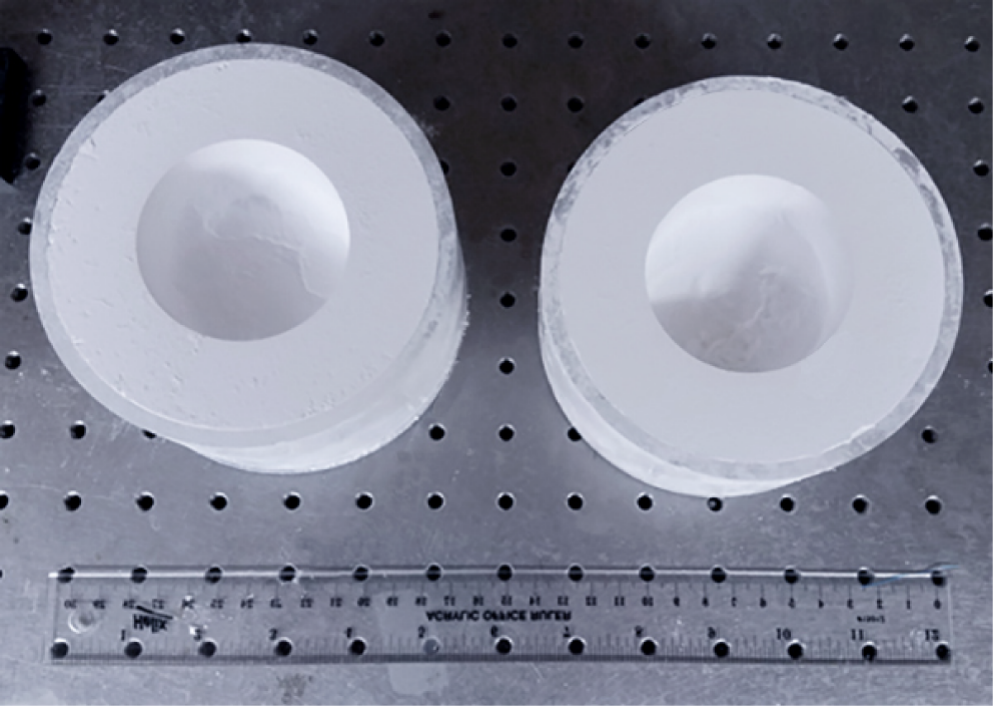
Right-cylindrical split-cavity HPIC fabricated from fumed silica packed within fused-quartz rings (101.6 mm inner diameter, 6.35 mm wall thickness, 50.8 mm height). The assembled internal cavity (50.8 mm in diameter and height) is split into two halves to facilitate sample placement and remains closed to prevent contamination. A 2–3 mm port in the upper half enables optical coupling for light delivery and collection. Adapted from author’s Dissertation: *Integrating Cavity Enhanced Vibrational Spectroscopy for Planetary Exploration*, Texas A&M University, 2025.^47^

The fumed silica is pressed into two fused quartz rings (6.35 mm wall thickness, 101.6 mm internal diameter, 50.8 mm height). A small port is drilled through the center of upper cavity half to couple light into and out of the cavity. The TAMU cavity employed a 2 mm diameter port, while the SwRI cavity has a 3 mm port. This split cavity design facilitates sample placement within the HPIC.

### Optical Configuration

For the iCERS Raman measurements, SwRI employed a 405 nm single longitudinal-mode laser coupled to the HPIC from which light was coupled out to a Horiba iHR320 spectrometer (Figure 3). The laser delivered 17 mW at the end of a 200 μm-core 0.22 NA multimode silica fiber (ThorLabs FG200UAE). The output end of the laser fiber was positioned inside the HPIC through the 3 mm-diameter port. A custom 400 μm-core 0.39 NA multimode fiber (ThorLabs FT400UMT) was carefully inserted into the 3 mm port to couple light out of the cavity with the other end connectorized with an SMA905 fiber connector. Although this fiber-based coupling method was convenient, it was not optimal, as a substantial portion of the light was lost through the 3 mm port. A 3 mm fused silica lightguide would provide significantly improved collection efficiency.

**Figure 3:**
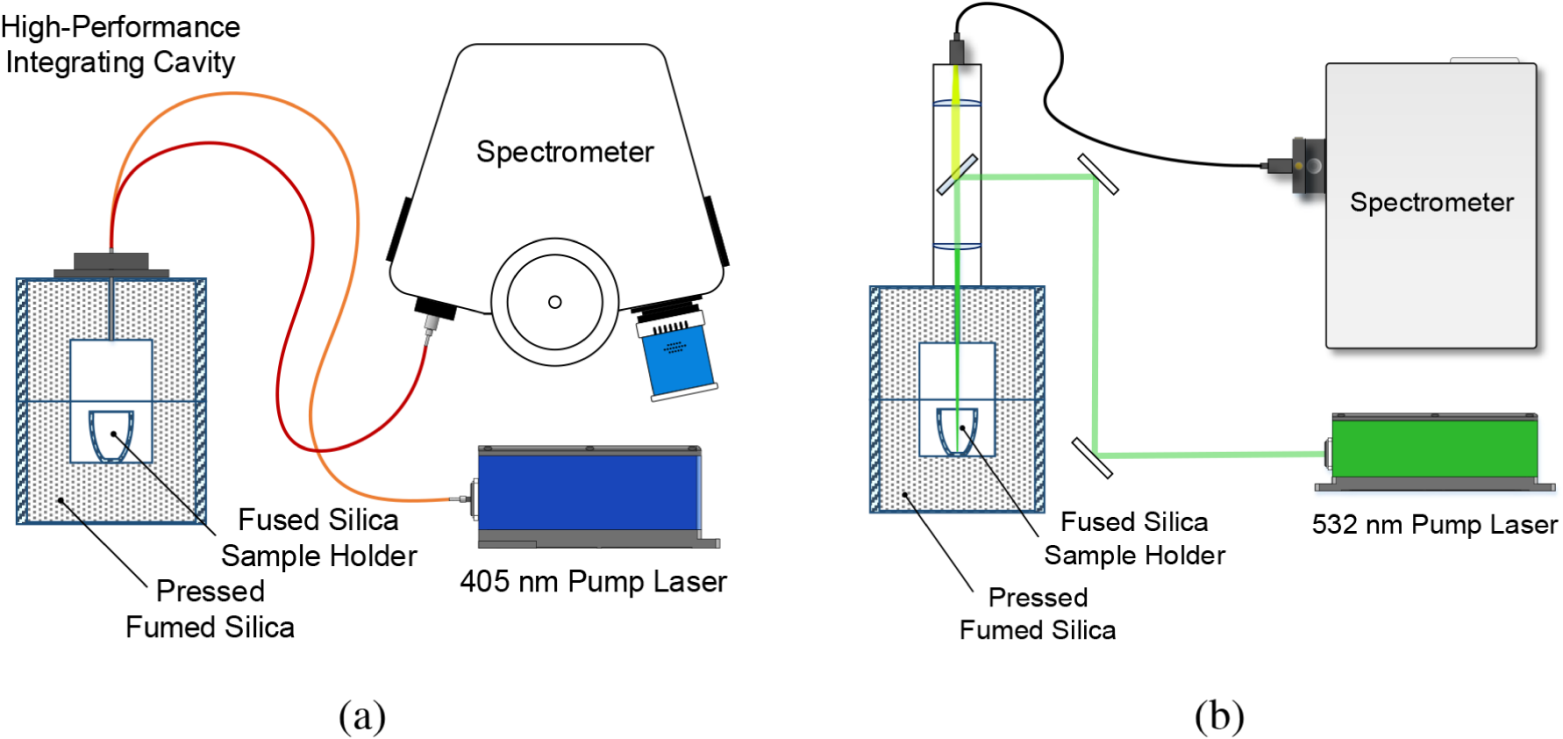
(a) SwRI iCERS setup for bulk liquids and solids: a 405 nm laser couples to a split-cavity HPIC via a 200 μm multimode fiber. Samples, in clean fused silica crucibles, are placed inside the HPIC. Light is collected via a 400 μm multimode fiber is coupled to a fiber collimator, long-pass filtered, and weakly focused through the entrance slit of a Horiba iHR320 spectrometer. Raman spectra are recorded on a Horiba Synapse CCD. Adapted from author’s Dissertation: *Integrating Cavity Enhanced Vibrational Spectroscopy for Planetary Exploration*, Texas A&M University, 2025.^47^ (b) TAMU iCERS setup for limit-of-detection studies: a 532 nm laser is free-space coupled onto a long-pass dichroic beam splitter into the HPIC and onto the sample. Extracted light is collimated through the dichroic, long-pass filtered, and focused into a fiber bundle coupled to an Acton Research Inspectrum300 modular spectrometer.

The output fiber was coupled to an SMA fiber collimator (ThorLabs F671SMA-405), long-pass filtered (Semrock LP02-407RU-12.5), and weakly focused through the 50 μm slit of a Horiba iHR320 spectrometer. The spectrometer was configured with a 1200 ln mm^–1^ grating with a 500 nm blaze angle and a 1024 × 256 front-illuminated open electrode Synapse charge-coupled device (Horiba Synapse 1024 × 256 FIOP model 354308) with a spectral resolution of 6.27 cm^1^. The Synapse detector is thermo-electrically cooled to −45 °C achieving a quantum efficiency of approximately 27% at 405 nm, increasing to approximately 33% at 500 nm.

A similar optical arrangement was used at TAMU, where a 150 mW 532 nm diode-pumped solid-state laser served as the pump source. The beam was free-space coupled through a periscope and reflected by a 532 nm dichroic long-pass filter (Semrock Di02-R532-25 × 36) into the HPIC. A 25.4 mm-diameter, 20 mm focal length aspheric condenser lens (ThorLabs ACL2520, 0.542 NA) focused the beam into the cavity and maximized light collection from the 2 mm port. The dichroic filter passes the Stokes scattered Raman light exiting the cavity before passing through another long-pass filter (Semrock BLP01-532-25). The light is then coupled into a fiber bundle using a matching 20 mm focal length aspheric condenser lens. The fiber bundle is coupled into an Acton Research Corporation InSpectrum300 spectrometer (10 μm slit. 1200 ln mm^–1^ grating, 500 nm blaze) equipped with a 1024 × 244 back-illuminated, cooled CCD detector array providing a resolution of 3.25 cm^–1^. The CCD detector array was cooled to −70 °C with a quantum efficiency of approximately 80% at 532 nm, increasing to approximately 93% at 650 nm.

### iCERS Measurements

The iCERS performance was evaluated by measuring Raman spectra of bulk MeOH, MgSO_4_, and glycine (Gly, CAS 54-40-6), and comparing them to reference spectra from the well-known databases. The samples were prepared as follows: MeOH (1.000 ± 0.0005 g, 31.210 mmol), MgSO_4_ (0.520 ± 0.0005 g, 4.320 mmol), and Gly (0.300 ± 0.0005 g, 3.996 mmol) were each transferred to a clean fused silica crucible and measured inside the HPIC. The fused silica crucible was thoroughly cleaned between measurements, and each cleaned crucible containing a new sample was carefully placed inside the integrating cavity to minimize the risk of contamination. The split cavity design allowed convenient sample placement while minimizing contamination by keeping the cavity closed during measurements and when not actively placing a sample inside.

### iCERS Limit of Detection Study

Detection limits were evaluated for two polycyclic aromatic hydrocarbons (PAHs): benzo­[a]­pyrene (BaP) and pyrene. BaP, a principal carcinogen in cigarette smoke, is classified as a Group 1 carcinogen by the U.S. Environmental Protection Agency,^49,50^ While pyrene is less toxic but environmentally hazardous.^51^

Samples were carefully weighed out into quartz crucibles using a microbalance (Citizen CX265): BaP (80–400 μg) and Pyrene (100–510 μg). These values were converted to a molar concentration by calculating the molar mass of each sample and dividing this by the volumetric area of the cavity bore. To demonstrate repeatability, five sets of Raman spectra were collected for each concentration. Integration times were set just below CCD saturation and normalized by scaling each oxygen (O_2_) peak of an individual spectrum to the mean intensity value of the O_2_ peak at each concentration.

## Results and Discussion

### Performance Characterization of Fumed-Silica HPICs

Ringdown measurements were obtained by SwRI for a newly fabricated right-cylindrical fumed-silica, split-cavity HPIC (internal cavity: 50.8 mm diameter × 54 mm height). Shortly after fabrication (September 9, 2021), the characteristic ringdown time at 610 nm was measured to be 202.1 ± 0.4 ns, (Figure 4a), corresponding to an average cavity reflectivity of 99.943 ± 0.0004%—to our knowledge, the highest reflectivity ever reported for a Lambertian material. Figure 4b shows the average cavity reflectivity from 248 to 1080 nm, where the reflectivity is significantly higher than commercially available Spectralon integrating cavities.^52^ In addition to the higher reflectivity in the visible to near-infrared spectral region, fumed silica HPICs maintain high reflectance into the ultraviolet (UV) and deep-UV spectral regions.

**Figure 4:**
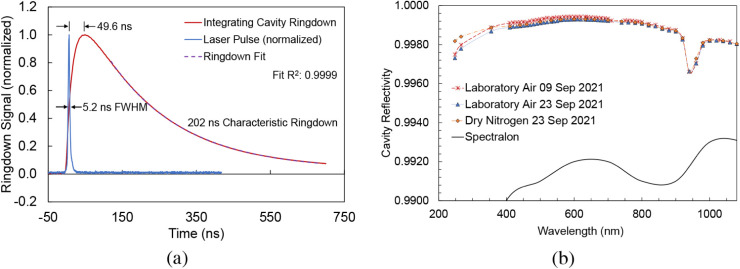
(a) HPIC ringdown signal collected on September 9, 2021, from a 5.2 ns full width at half-maximum input pulse at 610 nm. The characteristic ringdown time was found to be 202.1 ns corresponding to an average cavity reflectivity, *ρ̅*, of 99.94 ± 0.0004%. (b) Integrating cavity reflectivity from 248 to 1080 nm, determined from ringdown measurements. The published reflectivity for a Spectralon is shown for reference.^52^ The error of the calculated cavity reflectivity lies within the point. Adapted from author’s Dissertation: *Integrating Cavity Enhanced Vibrational Spectroscopy for Planetary Exploration*, Texas A&M University, 2025.^47^

Additional ringdown measurements were obtained 14 days later (September 23, 2021) after exposure to the atmosphere and laboratory environment resulted in a slight decrease in reflectivity below 610 nm (Figure 4b). The reflectivity above 610 nm remained largely unchanged. The observed degradation arises from the large surface area of the fumed silica, which readily absorbs moisture and attracts contaminants such as dust and pollen from the air. Consequently, HPICs open to the atmosphere degrade over time, with a typical laboratory service life of 6–9 months, depending on the environmental conditions. Volatile analytes (e.g., acetic acid) can also be absorbed, leading to contamination. Contaminated cavities can be recovered by baking the cavity in a furnace, or vacuum furnace, up to 900 °C. Alternatively, sealed HPIC designs that environmentally isolate the fumed silica between a sealed body and internal fused-silica cell eliminate environmental contamination.^53,54^ The internal fused-silica cell can be readily cleaned using conventional laboratory cleaning procedures.

The effective optical path length, *l_eff_* = *n̅* · *d̅*, of the cavity was 52.3 m, with the absorption in air significantly impacting the integrating cavity performance. Ringdown measurements collected under ambient air and dry nitrogen (N_2_) (Figure 4b) showed that N_2_ purging improved reflectivity shortward of 500 nm and markedly enhanced performance at 355, 266, and 248 nm due to reduced absorption. These results confirm that fumed-silica HPICs maintain excellent optical performance into the deep-UV regime.

### Background Characterization and iCERS Performance

A background spectrum acquired from the empty HPIC (Figure 5a) was collected for a 6 s integration time over 100 acquisitions (total 600 s). Prominent Raman peaks from fumed and fused silica were observed at 412 cm^1^ (*ω*_1_), 467 cm^–1^ (*D*_1_), 603 cm^–1^ (*D*_2_), 779 cm^–1^ (*ω*_3_), 1061 cm^–1^ (*ω*_4_,*TO*), and 1205 cm^–1^ (*ω*_5_,*LO*). Background measurements of an empty cavity in air reveal Raman peaks of N_2_ (2330 cm^1^) and O_2_ (1556 cm^–1^) were clearly identifiable. The fumed/fused silica Raman background, however, lies within the fingerprint region (300–1200 cm^–1^), complicating background removal. Despite the nonoptimized optical coupling, the system achieved high count rates. Comparable background spectra recorded at TAMU (Figure 5b) showed nearly identical profiles, confirming reproducibility. Importantly, the ability of iCERS to resolve gas-phase O_2_ and N_2_ from the ambient atmosphere represents a significant advance over conventional spontaneous Raman spectroscopy.

**Figure 5:**
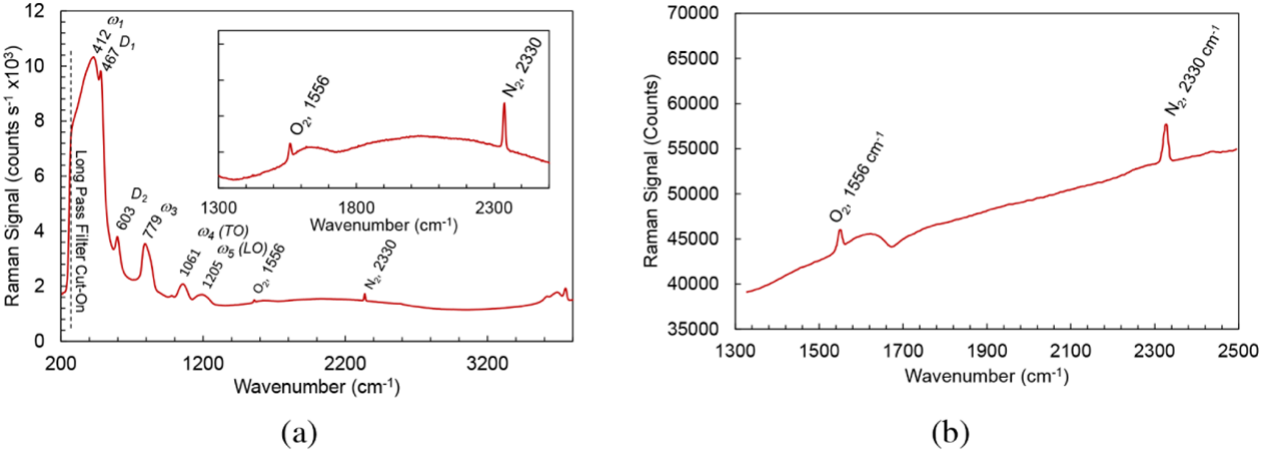
(a) Unprocessed Raman background spectrum from the SwRI HPIC. The long-pass cut-on is just below 300 cm^–1^, and primary Raman peaks are labeled. The background includes the laboratory air, where O_2_ (1556 cm^–1^) and N_2_ (2330 cm^–1^) are clearly identifiable. Adapted from author’s Dissertation: *Integrating Cavity Enhanced Vibrational Spectroscopy for Planetary Exploration*, Texas A&M University, 2025.^47^ (b) Unprocessed Raman background spectrum of lab air from the TAMU HPIC. The O_2_ and N_2_ peaks closely match the data collected by SwRI. Adapted from coauthor’s Dissertation: *Integrating Cavity Enhanced Spectroscopy for Liquid and Gas Sensing*, Texas A&M University, 2015.^48^

Direct comparisons between iCERS and SCRS were performed for laboratory air, bulk MeOH, and crystalline MgSO_4_ samples. Atmospheric O_2_ (1556 cm^–1^) and N_2_ (2330 cm^–1^) peaks were readily identifiable in the background (Figure 5). The peak differential Raman signal count rate for N_2_ was 4401 counts s^–1^ with iCERS, compared to 0.027 counts s^–1^ obtained with SCRS (Figure 6). The noise standard deviation was 31.74 counts s^–1^ for iCERS and 0.003 counts s^–1^ for the SCRS measurement, yielding signal-to-noise ratios (SNRs) of 139:1 and 10:1, respectively. The O_2_ band at 1556 cm^–1^ was readily resolved in the iCERS spectrum but was indistinguishable from noise in the SCRS measurement. While SCRS is inherently limited for gas-phase measurements, these results demonstrate how the increased photon–molecule interaction within the HPIC volume dramatically enhances Raman signal intensity.

**Figure 6:**
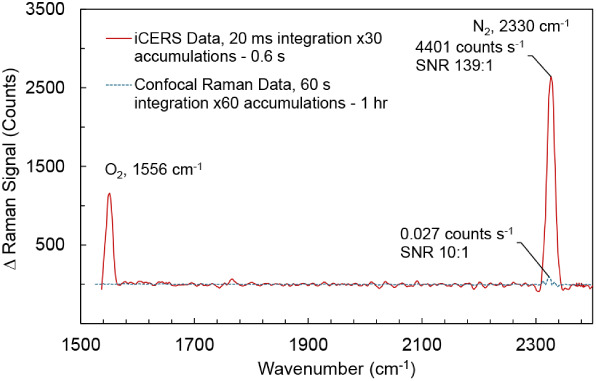
Direct comparison of differential Raman signals of laboratory air obtained using iCERS and SCRS.^48^ The iCERS data was acquired with a 20 ms integration over 30 acquisitions (total 0.6 s), while SCRS data required a 60 s integration time over 60 accumulations (total 1 h). The peak N_2_ Raman line count rate was 4401 counts s^–1^ for iCERS and 0.027 counts s^–1^ for SCRS, yielding signal-to-noise ratios (SNRs) of 139:1 and 10:1, respectively. The O_2_ Raman line was undetectable for SCRS. Adapted from coauthor’s Dissertation: *Integrating Cavity Enhanced Spectroscopy for Liquid and Gas Sensing*, Texas A&M University, 2015.^48^

Comparative measurements of bulk liquid and solid samples further validated iCERS performance. Both SCRS and iCERS yielded clean, well-resolved Raman spectra (Figure 7). For 1 mL of MeOH, the characteristic Raman peaks—CO stretch (1037 cm^–1^), CH_3_ bend (1453 cm^–1^), CH_3_ symmetric and asymmetric stretches (2836 and 2946 cm^–1^), and OH stretch (3350 cm^–1^)—were observed in both configurations. The differential Raman signal count rate for the CH_3_ symmetric stretch was 5683 counts s^–1^ for iCERS and 394 counts s^–1^ for SCRS. The noise standard deviation was 4.30 counts s^–1^ for iCERS and 0.98 counts s^–1^ for SCRS measurements, yielding a SNR of 1322 and 402 for iCERS and SCRS respectively. For crystalline MgSO_4_ (520 mg), the SO_4_^2–^ symmetric stretch (∼1000 cm^–1^) exhibited differential count rates of 2626 counts s^–1^ for iCERS and 338 counts s^–1^ for SCRS. The noise standard deviation was 1.78 counts s^–1^ for iCERS and 1.11 counts s^–1^ for SCRS measurements, yielding a SNR of 1478 and 291 for iCERS and SCRS respectively.

**Figure 7:**
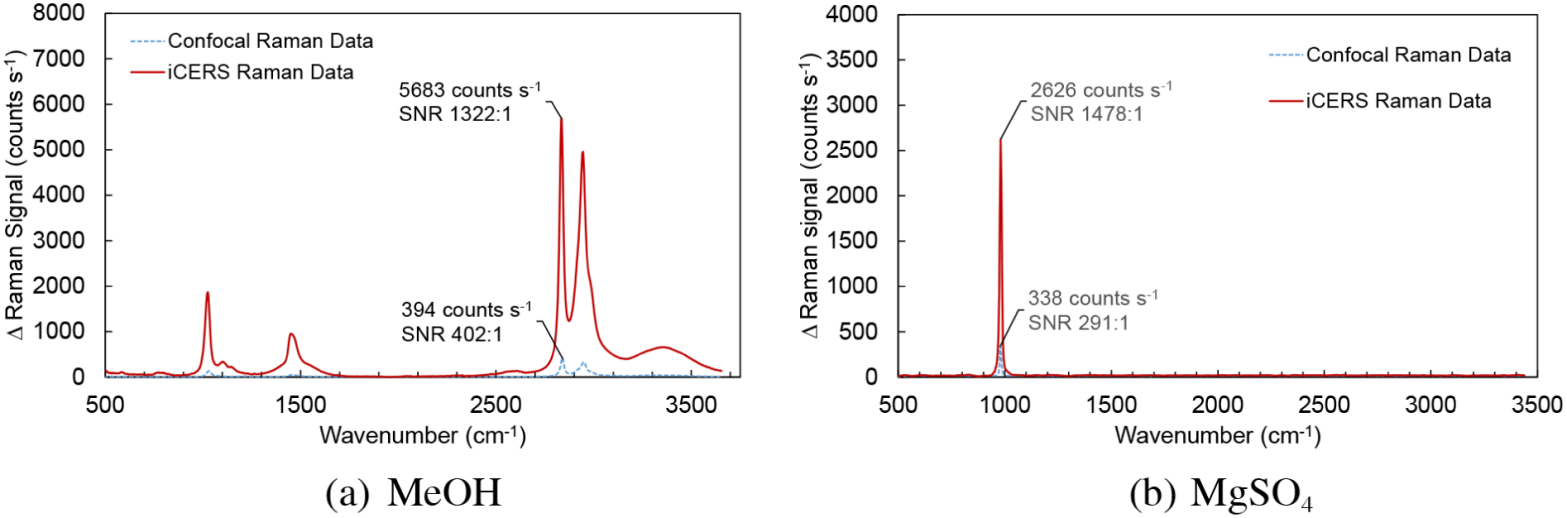
Direct comparison of differential Raman measurements using iCERS and SCRS at 405 nm and 20 °C: (a) liquid MeOH (1 mL) CH_3_ symmetric stretch: 5683 counts s^–1^ for iCERS versus 394 counts s^–1^ for SCRS and (b) MgSO_4_ crystals (520 mg) symmetric stretch: 2626 counts s^–1^ for iCERS versus 338 counts s^–1^ for SCRS. Enhancement factors depend on sample size, distribution, and optical properties. Adapted from author’s Dissertation: *Integrating Cavity Enhanced Vibrational Spectroscopy for Planetary Exploration*, Texas A&M University, 2025.^47^

While iCERS demonstrated significant enhancement for the gaseous, liquid, and solid samples, the enhancement magnitude varied due to differences in sample absorption and distribution. Optimizing the sample size and distribution within the HPIC can further increase iCERS enhancement. Nevertheless, iCERS provides significant signal enhancement even for unprocessed bulk samples, underscoring its robustness and versatility for a wide range of analytical applications.

In SCRS, laser power is often limited by the sample’s photodamage threshold. In the comparative measurements reported here, the laser power was unchanged. Within the HPIC, however, the sample irradiance remains low, on the same order as the input laser power (*ϕ*_0_) magnitude where *M*/*πS* ∼ 1 cm^–2^ sr^–1^. Consequently, laser power can be increased substantially without inducing photodamage. Because Raman signal intensity scales linearly with incident power, iCERS can achieve even greater enhancement using high-power continuous-wave or high-peak-power pulsed lasers.

### iCERS Background Subtraction

A notable complexity unique to iCERS is the intrinsic Raman background from the HPIC (Figure 5). While background interference is a well-known challenge in Raman spectroscopy,^55^ the fumed silica background below 700 cm^–1^ is particularly strong. However, several computational approaches for background removal are available including deconvolution and baseline correction.^56–60^ Shifted-excitation Raman difference spectroscopy^61^ is another method that can be used to mitigate strong fluorescence background that is well suited to iCERS.

In this work, an iterative background subtraction routine was implemented in MATLAB. Prior to sample measurement, a background spectrum was acquired and an automated fit model of the background was generated. During sample analysis, the background fit model was fitted to the sample data using the characteristic background Raman peaks and subtracted to yield the differential Raman signal. When necessary, baseline correction and low-pass filtering were applied to minimize residual noise and high-frequency artifacts. This approach produced clean, reproducible differential Raman spectra while maintaining quantitative fidelity.

### iCERS Measurements

Raman spectra of bulk MeOH, MgSO_4_, and Gly were acquired using a Horiba iHR320 spectrometer. The MeOH and Gly spectra showed excellent agreement with reference data from the National Institute of Advanced Industrial Science and Technology (AIST) Spectral Database for Organic Compounds (SDBS).^62^ The 488 nm reference spectra are well suited for comparison with 405 nm iCERS measurements. Similarly, MgSO_4_ spectra correspond closely with data from the RRUFF mineral database.^63^

### MeOH Analysis

Figure 8a displays the MeOH Raman spectrum obtained using the iCERS technique. The MeOH sample was measured as a bulk liquid at 20 °C. All four prominent spectral peaks, including the O–H stretch mode, were clearly identifiable. Spectra were acquired with a 3 s integration time over 100 accumulations. After background subtraction, the noise standard deviation was 2.33 counts s^–1^. Based on the peak intensity, the noise-equivalent detection limit for MeOH was estimated to be 12.85 μmol for the 300 s acquisition.

**Figure 8:**
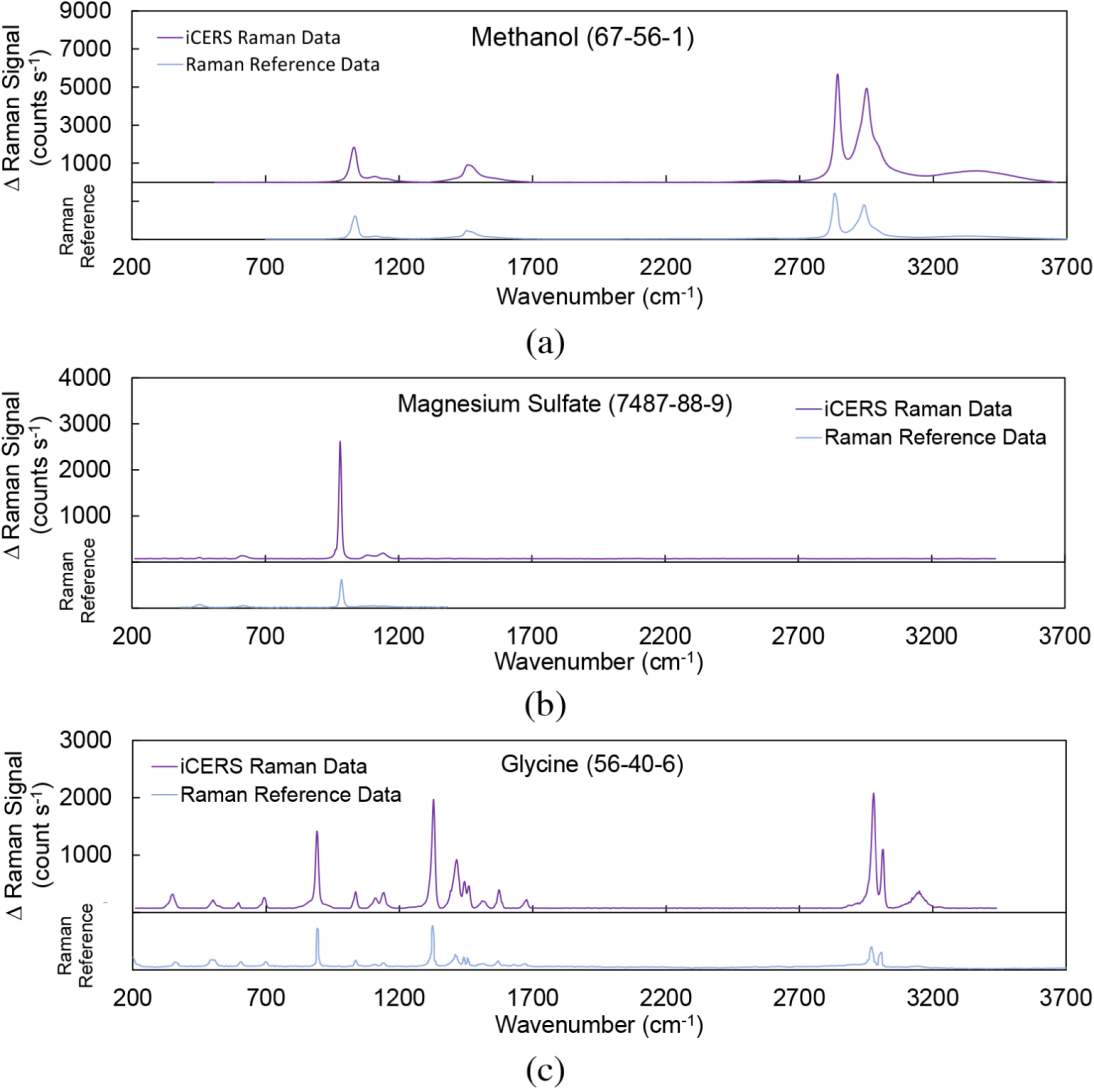
Differential Raman measurements using iCERS system at 405 nm and 20 °C. (a) Liquid MeOH, 3 s integration over 100 acquisitions, closely matches the SDBS Raman reference with a 12.85 μmol noise-equivalent detection limit; (b) Crystalline MgSO_4_, 6 s integration over 100 acquisitions, matches the RRUFF Raman reference with a 2.58 μmol detection limit; and (c) Gly powder, 6 s integration over 100 acquisitions, matches the SDBS Raman reference database with a 3.49 μmol detection limit. Adapted from author’s Dissertation: *Integrating Cavity Enhanced Vibrational Spectroscopy for Planetary Exploration*, Texas A&M University, 2025.^47^

### MgSO_4_ Analysis

Figure 8b shows the MgSO_4_ Raman spectrum collected using iCERS. The bulk crystalline powder was measured at 20 °C. Magnesium sulfate exhibits several polymorphic and hydrated forms, distinguishable by shifts in the SO_4_^2–^ symmetric stretch near 1000 cm^–1^. The dominant peak observed at 986 cm^–1^ indicates a hydrated form, and comparison with the RRUFF database suggests that the sample is epsomite (MgSO_4_·7H_2_O). The measurement was acquired with a 6 s integration over 100 accumulations. After background subtraction, the noise standard deviation was 1.52 counts s^–1^, yielding a noise-equivalent detection limit of 2.58 μmol for MgSO_4_ for the 600 s acquisition.

### Gly Analysis

Figure 8c shows the Gly Raman spectrum collected using iCERS. A 3.996 mmol Gly sample was measured as bulk powder at 20 °C. The spectrum closely matches the corresponding SDBS reference data. Prominent peaks at 895, 1037, 1327, 1413, 1442, 1456, 2973, and 3009 cm^–1^ were clearly identified. Spectra were recorded using a 3 s integration time over 100 accumulations. After background subtraction, the noise standard deviation was 1.75 counts s^–1^, corresponding to a noise-equivalent detection limit of 3.49 μmol for Gly for the 300 s acquisition.

### PAH Limit of Detection Studies

Limit-of-detection studies for two polycyclic aromatic hydrocarbons (PAHs), benzo­[a]­pyrene (BaP) and pyrene, were performed at TAMU. Raman spectra of the PAHs were obtained using a custom-built Raman microscope to serve as reference data for the iCERS measurements. Reference Raman spectra acquired with 532 nm excitation for BaP and pyrene are shown in Figure 9a and Figure 9b, respectively. The strongest Raman shift for BaP occurred at 1235 cm^–1^, with a secondary line at 1385 cm^–1^, both of which were used to determine detection limits. Pyrene exhibited its strongest Raman band at 1403 cm^–1^, accompanied by a secondary peak at 1239 cm^–1^; the 1403 cm^–1^ band was primarily used for quantitative analysis. All samples were measured in clean fused silica crucibles.

**Figure 9:**
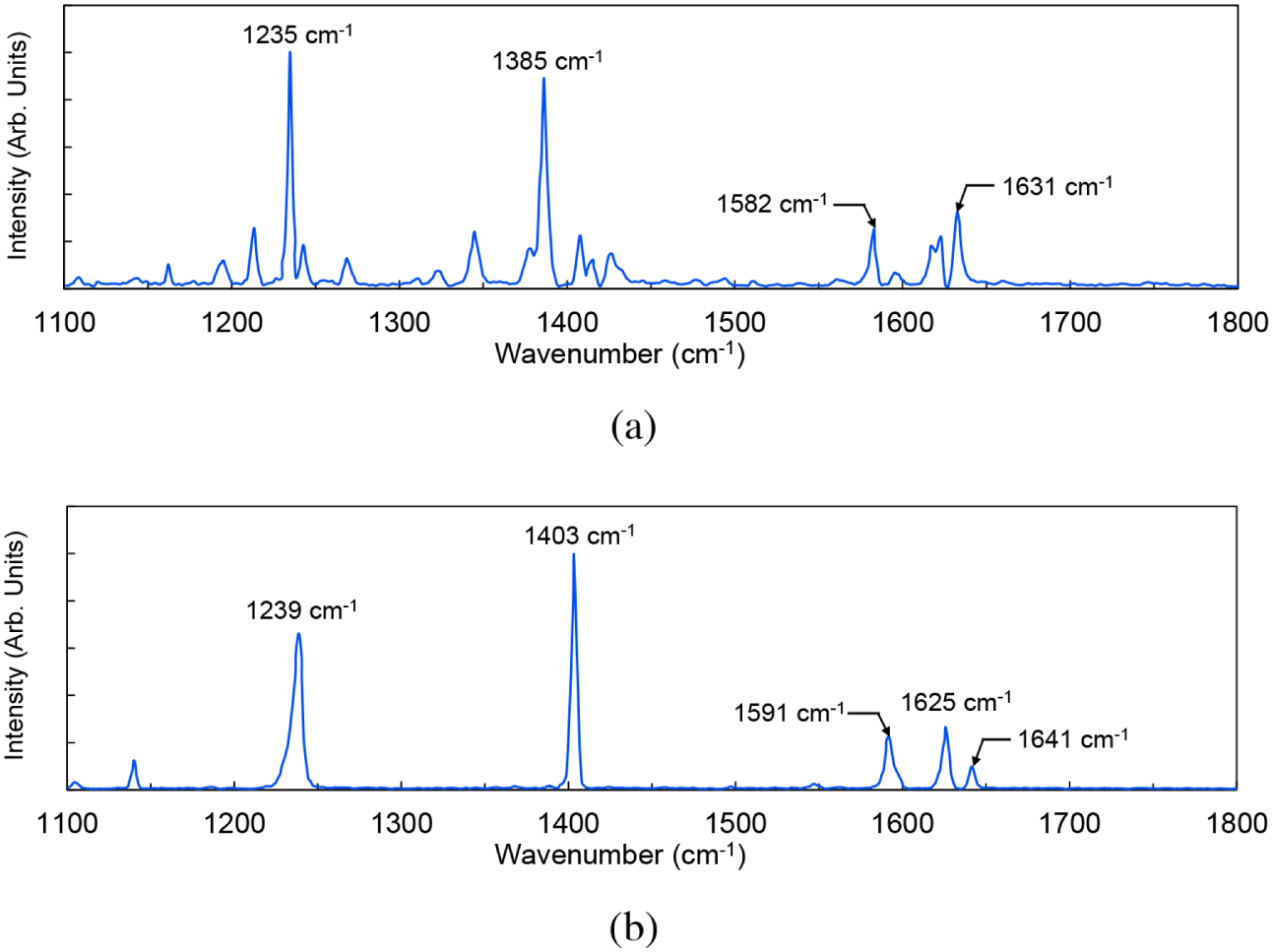
Raman reference spectra recorded using a Horiba Scientific Raman microscope: (a) BaP; and (b) pyrene. The Raman shifts for key lines are labeled. Adapted from coauthor’s Dissertation: *Integrating Cavity Enhanced Spectroscopy for Liquid and Gas Sensing*, Texas A&M University, 2015.^48^

To ensure spectral consistency, the O_2_ and N_2_ Raman peaks were used for normalization between samples, as the intensities of these lines remain constant across measurements. This approach also compensates for small variations in cavity alignment and allows scaling of spectra collected under different integration times.

### BaP Quantification

Figure 10a displays the 532 nm Raman spectra for all BaP concentrations after background subtraction. The intensity of the 1385 cm^–1^ Raman band was used to generate a concentration–intensity calibration curve (Figure 10b). This band was selected due to its relative strength and proximity to the O_2_ peak. Background subtraction was performed using an iterative cubic spline fitting routine.^48^ Linear regression of the Raman intensity versus BaP concentration yielded R^2^ = 0.933. Error bars represent the standard deviation of the mean peak intensity for five replicates. The minimum concentration was constrained by the smallest measurable mass on the analytical balance. From the regression equation, the detection limit for BaP under 532 nm excitation was determined to be 700 nM (72 nmol BaP).^48^

**Figure 10:**
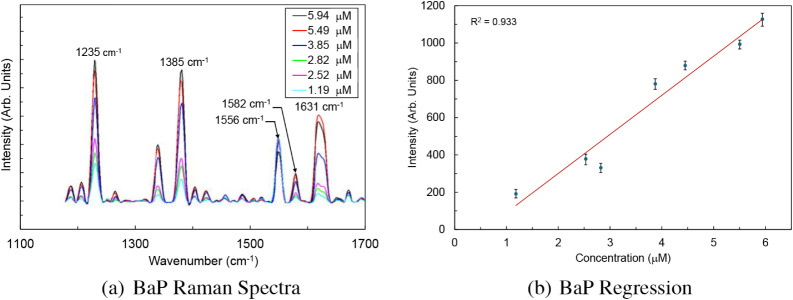
(a) Raman spectra at 532 nm for BaP measured inside an integrating cavity with background subtraction. (b) Regression of the intensity of the 1385 cm^–1^ Raman line versus the amount of BaP in the integrating cavity. A linear regression fit to this data resulted in R^2^ = 0.933 with a detection limit for BaP of 700 nM (72 nmol BaP) with 532 nm excitation. Adapted from coauthor’s Dissertation: *Integrating Cavity Enhanced Spectroscopy for Liquid and Gas Sensing*, Texas A&M University, 2015.^48^

### Pyrene Quantification

Figure 11a shows the 532 nm Raman spectra for all pyrene concentrations after background subtraction. The 1403 cm^–1^ Raman band was used for quantitative analysis, and the resulting calibration plot is shown in Figure 11b. Linear regression produced R^2^ = 0.975, indicating excellent linearity. The minimum detectable concentration for pyrene at 532 nm was 690 nM (71 nmol Pyrene).^48^

**Figure 11:**
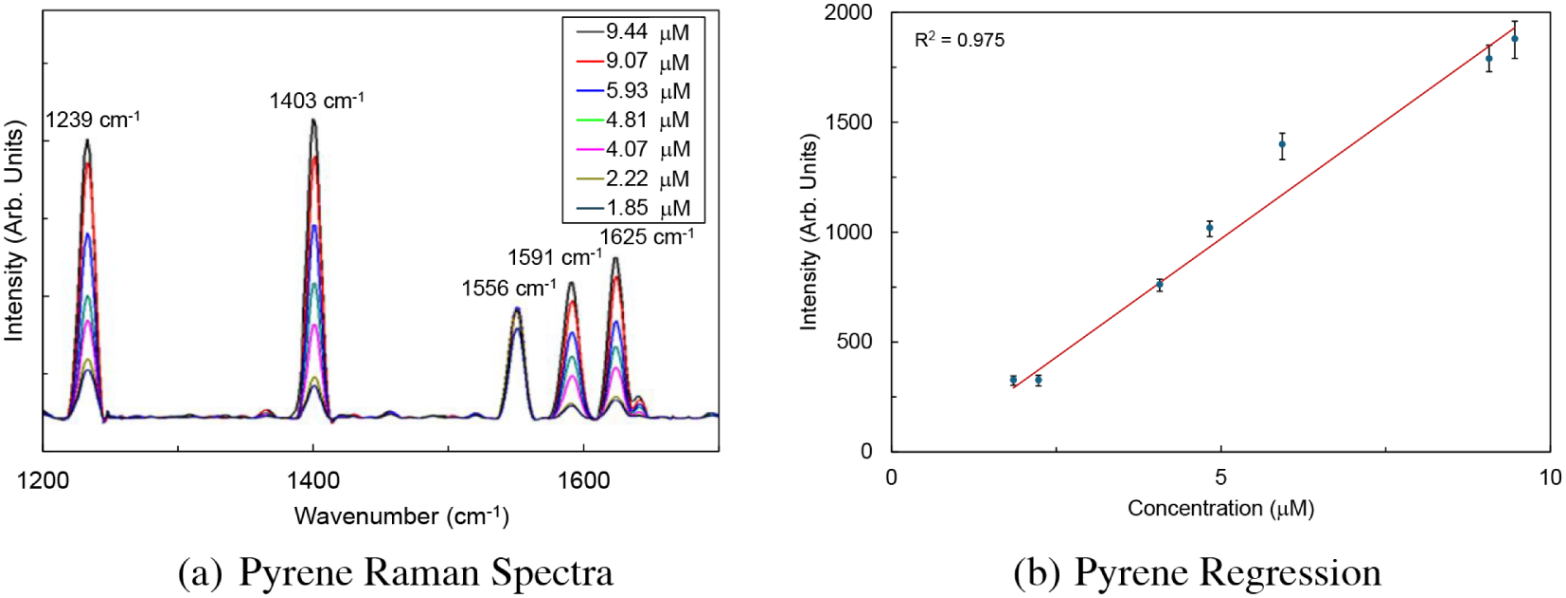
Raman spectra at 532 nm of pyrene measured inside an integrating cavity with background subtraction. (b) Regression of the intensity of the 1403 cm^–1^ Raman line vs the amount of pyrene in the integrating cavity. A linear regression fit to this data resulted in R^2^ = 0.975 with a detection limit for pyrene of 690 nM (71 nmol Pyrene) with 532 nm excitation. Adapted from coauthor’s Dissertation: *Integrating Cavity Enhanced Spectroscopy for Liquid and Gas Sensing*, Texas A&M University, 2015.^48^

The limit of detection studies demonstrate that iCERS enables sensitive analyte detection. Further limit of detection studies, reported by Bixler^48^ for Pyrene at 473 nm, using a 3*σ* detection threshold, yielded a 13.16 nM detection limit. iCERS offers a powerful analytical approach that combines excellent detection sensitivity with reliable quantitative capabilities.

## Conclusions

In conclusion, we have demonstrated that the iCERS technique can perform Raman measurements of both bulk liquids and solids with significant enhancement compared to conventional SCRS. The enhancement was achieved without optimization of sample size or spatial distribution, highlighting the robustness of iCERS and its minimal sample preparation requirements. In addition to bulk sample analysis, iCERS also enabled simultaneous detection of atmospheric gases. The observed enhancement is expected to increase further for optimized sample size and distribution due to reduced absorption losses and a greater number of internal reflections.

Limit-of-detection analyses confirmed the quantitative capability of the technique, achieving detection limits of 700 nM (72 nmol BaP) for benzo­[a]­pyrene and 690 nM (71 nmol Pyrene) for pyrene using 532 nm excitation. Reflectivity measurements of the integrating cavity show that iCERS can also operate in the UV and deep-UV regions, where resonance Raman scattering (RRS) can provide additional enhancement while mitigating fluorescence interference. Furthermore, because iCERS enhances spontaneous Raman scattering without modifying vibrational line shapes, spectral distortion and peak shifting are not a concern. The technique’s inherently low sample irradiance allows the use of higher power or high peak power lasers without inducing photodamage, further improving signal strength.

Overall, these results demonstrate the versatility and efficacy of iCERS for sensitive, quantitative Raman measurements of bulk compounds and gases. Future integration of surface-enhanced Raman scattering (SERS) substrates within the cavity could potentially extend this technique to single-molecule detection.

## §

Sextant Strategy LLC, 24393 Rock Pond Ct, Aldie, VA 20105, United States

## ∥

Air Force Research Laboratory, JBSA Fort Sam Houston, Texas 78234, United States

## ⊥

EO Solutions, 147C Westchester Rd, Madison, Alabama 35758, United States

## #

U.S. Army Space and Missile Defense Command, Huntsville, Alabama 35898, United States
